# Correlation of Immunological and Molecular Profiles with Response to Crizotinib in Alveolar Soft Part Sarcoma: An Exploratory Study Related to the EORTC 90101 “CREATE” Trial

**DOI:** 10.3390/ijms23105689

**Published:** 2022-05-19

**Authors:** Che-Jui Lee, Elodie Modave, Bram Boeckx, Bernd Kasper, Steinar Aamdal, Michael G. Leahy, Piotr Rutkowski, Sebastian Bauer, Maria Debiec-Rychter, Raf Sciot, Diether Lambrechts, Agnieszka Wozniak, Patrick Schöffski

**Affiliations:** 1 Laboratory of Experimental Oncology, Department of Oncology, KU Leuven, 3000 Leuven, Belgium; jerry.lee@kuleuven.be (C.-J.L.); agnieszka.wozniak@kuleuven.be (A.W.); 2 VIB Center for Cancer Biology, VIB and Department of Human Genetics, KU Leuven, 3000 Leuven, Belgium; elodie.modave@kuleuven.be (E.M.); bram.boeckx@kuleuven.be (B.B.); diether.lambrechts@kuleuven.be (D.L.); 3 Sarcoma Unit, Interdisciplinary Tumor Center, Mannheim University Medical Center, 68167 Mannheim, Germany; bernd.kasper@medma.uni-heidelberg.de; 4 Department of Oncology, Oslo University Hospital, 0315 Oslo, Norway; steinar.aamdal@medisin.uio.no; 5 The Christie NHS Foundation Trust, Manchester M20 4BX, UK; michael.leahy2@nhs.net; 6 Department of Soft Tissue/Bone Sarcoma and Melanoma, Maria Sklodowska-Curie National Research Institute of Oncology, 00-001 Warsaw, Poland; piotr.rutkowski@pib-nio.pl; 7 German Cancer Consortium (DKTK), Partner Site University Hospital Essen, 45147 Essen, Germany; sebastian.bauer@uk-essen.de; 8 Department of Human Genetics, University Hospitals Leuven, KU Leuven, 3000 Leuven, Belgium; maria.debiec-rychter@kuleuven.be; 9 Department of Pathology, University Hospitals Leuven, KU Leuven, 3000 Leuven, Belgium; raf.sciot@uzleuven.be; 10 Department of General Medical Oncology, Leuven Cancer Institute, University Hospitals Leuven, KU Leuven, 3000 Leuven, Belgium

**Keywords:** alveolar soft part sarcoma, immunological characterization, tumor microenvironment, molecular profiling, gene alteration, crizotinib, CREATE

## Abstract

Alveolar soft part sarcoma (ASPS) is a rare subtype of soft tissue sarcoma characterized by an unbalanced translocation, resulting in ASPSCR1-TFE3 fusion that transcriptionally upregulates *MET* expression. The European Organization for Research and Treatment of Cancer (EORTC) 90101 “CREATE” phase II trial evaluated the MET inhibitor crizotinib in ASPS patients, achieving only limited antitumor activity. We performed a comprehensive molecular analysis of ASPS tissue samples collected in this trial to identify potential biomarkers correlating with treatment outcome. A tissue microarray containing 47 ASPS cases was used for the characterization of the tumor microenvironment using multiplex immunofluorescence. DNA isolated from 34 available tumor samples was analyzed to detect recurrent gene copy number alterations (CNAs) and mutations by low-coverage whole-genome sequencing and whole-exome sequencing. Pathway enrichment analysis was used to identify diseased-associated pathways in ASPS sarcomagenesis. Kaplan–Meier estimates, Cox regression, and the Fisher’s exact test were used to correlate histopathological and molecular findings with clinical data related to crizotinib treatment, aiming to identify potential factors associated with patient outcome. Tumor microenvironment characterization showed the presence of PD-L1 and CTLA-4 in 10 and 2 tumors, respectively, and the absence of PD-1 in all specimens. Apart from CD68, other immunological markers were rarely expressed, suggesting a low level of tumor-infiltrating lymphocytes in ASPS. By CNA analysis, we detected a number of broad and focal alterations. The most common alteration was the loss of chromosomal region 1p36.32 in 44% of cases. The loss of chromosomal regions 1p36.32, 1p33, 1p22.2, and 8p was associated with shorter progression-free survival. Using whole-exome sequencing, 13 cancer-associated genes were found to be mutated in at least three cases. Pathway enrichment analysis identified genetic alterations in NOTCH signaling, chromatin organization, and SUMOylation pathways. NOTCH4 intracellular domain dysregulation was associated with poor outcome, while inactivation of the beta-catenin/TCF complex correlated with improved outcome in patients receiving crizotinib. ASPS is characterized by molecular heterogeneity. We identify genetic aberrations potentially predictive of treatment outcome during crizotinib therapy and provide additional insights into the biology of ASPS, paving the way to improve treatment approaches for this extremely rare malignancy.

## 1. Introduction

Alveolar soft part sarcoma (ASPS) is a very rare subtype of soft tissue sarcoma (STS) of uncertain histogenesis, with a yearly incidence of 1/10^7^ per year, predominantly involving deep soft tissue of the extremities and mainly affecting a very young population [1]. Although this tumor grows slowly, its prognosis is poor, and the incidence of distant metastasis is high, with a predilection of metastasis location in the lungs, brain, bones, and lymph nodes [2]. Chemotherapy is ineffective in the treatment of this orphan neoplasm, while radiotherapy may play a role in reducing the risk for local recurrences after surgery [3]. This tumor has morphological features comprising uniform nests of polygonal cells with well-defined cell borders, eosinophilic granular cytoplasm, and a rounded central nucleus. The nests of tumor cells typically appear in an alveolar pattern [4]. ASPS is characterized by a specific, unbalanced translocation der(17)t(X;17)(p11;p25), resulting in the formation of the alveolar soft part sarcoma critical region 1-transcription factor E3 (ASPSCR1-TFE3) fusion that transcriptionally upregulates *MET*, which leads to overexpression of the MET receptor [5]. MET inhibition was therefore suggested as a therapeutic approach for this ultra-rare tumor.

In the European Organization for Research and Treatment of Cancer (EORTC) phase II trial 90,101 “CREATE,” the MET inhibitor crizotinib was evaluated in patients with MET-positive and MET-negative ASPS. MET status was defined by the presence or absence of *TFE3* rearrangement by fluorescence in situ hybridization (FISH). Even though the vast majority of ASPS treated in the CREATE trial were classified as MET-positive, MET inhibition with the oral tyrosine kinase inhibitor was only associated with very sporadic responses. Nevertheless, the long duration of treatment (median number of three-weekly cycles: 12.5) and the very high disease control rate (90%) observed in patients with *TFE3*-rearranged tumors suggested some activity of crizotinib in this trial [6]. Interestingly, exceptional responses in two cases (one MET-positive, one MET-negative) suggested the contribution of other molecular factors in addition to *TFE3* rearrangement in ASPS development. Previous studies in MET-driven non-small cell lung cancer (NSCLC) linked *MET* exon 14-alterations with crizotinib sensitivity, while phosphoinositide 3-kinase (PI3K) pathway alterations correlated with a worse response to MET inhibition [7,8]. In addition to the genomic alterations, previous studies also revealed the presence of programmed cell death protein ligand 1 (PD-L1) and CD8-positive tumor-infiltrating lymphocytes (TILs) to be associated with a worse clinical outcome in epidermal growth factor receptor (*EGFR*) mutated NSCLC patients, receiving an EGFR inhibitor [9], indicating that the immunological composition of the tumor microenvironment (TME) could play a role in the response to tyrosine kinase inhibitors.

Interestingly, in contrast with the majority of other soft tissue sarcomas, ASPS is believed to be sensitive to immune checkpoint inhibitors (ICIs), as presented in several case reports and in a phase II trial of atezolizumab, in which 8 out of the 19 patients had durable responses [10,11]. However, it is still unclear why this specific STS subtype tends to respond to immunotherapy. The presence of immune checkpoints, TILs, and other immunological components in the TME have been reported to predict the clinical benefit on immunotherapy in other malignancies [12,13]. Therefore, a further study of the ASPS molecular profile and TME characterization is warranted and may direct better disease management for patients with this ultra-rare disease.

## 2. Results

### 2.1. Patient Cohort

Archival tumor material was available from 47 centrally confirmed ASPS patients. These samples included 43 MET-positive, three MET-negative, and one MET-unevaluable tumor according to the criteria of the original trial protocol. Biological material was composed of 37 primary tumors and 10 metastatic lesions. The male-to-female ratio was 1.24 and the median age at enrollment of the trial was 32 years (range: 16–69). Among 43 clinically evaluable patients, two had a partial response (PR), and 35 had stable disease (SD) as the best response to crizotinib. Progressive disease (PD) was the best response in 6 cases. Median progression-free survival (PFS) and overall survival (OS) were 6 and 27.1 months, respectively, for the evaluable patient populations. For subsequent analyses, 47 cases were included in the tissue microarray (TMA) and therefore, were available for histopathological evaluation. DNA samples were isolated from 34 cases and subjected to an extensive molecular genetic analysis (other samples were either limited or of low quality). Clinicopathological variables for all individual patients are summarized in Table 1. 

### 2.2. Characterization of Immunological Components in the Tumor Microenvironment

**Figure 1:**
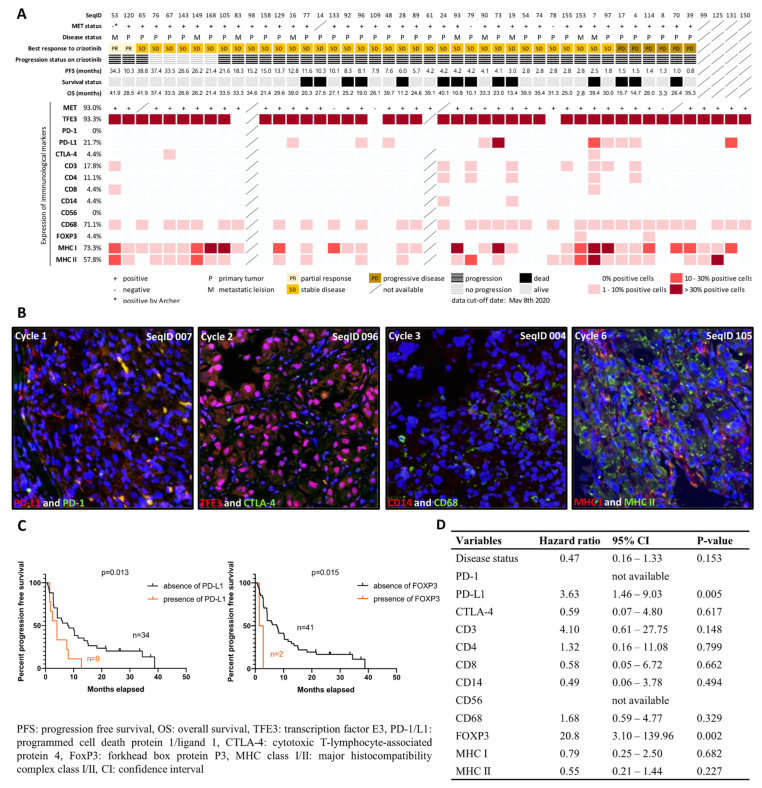
Overview and representative images for characterization of tumor microenvironment in alveolar soft part sarcoma. (**A**) The heatmap demonstrates an overview of the expression of the immunocompetent components in the tumor, tested across 47 cases on a tissue microarray. The MET expression was determined by immunohistochemical positivity. The expression level was determined by the percentage of immunopositive cells among cores per case, and the mean was recorded as the final result. A MET-negative case labeled with * was subjected to the *post hoc* Archer analysis and identified as a *TFE3*-rearranged tumor. (**B**) Representative images show immunofluorescence-stained tissue in different cycles of the MILAN procedure, with different markers. Images were digitally scanned using 200-fold magnification. The blue color showed 4′,6-diamidino-2-phenylindole (DAPI) staining and represented nucleated cells. Membrane expression of programmed cell death ligand 1 (red) was detected in a small proportion of tumor cells in the first cycle; no expression for programmed cell death 1 (green) was observed. In the second cycle, pink color on merged images indicated nuclear localization of transcription factor E3 (red), but no green fluorescence for cytotoxic T lymphocyte-associated protein 4 was detected. In the third cycle, membrane expression of CD14 (red) and CD68 (green) were detected. The presence of major histocompatibility complex class I (red) and II (green) was demonstrated in the sixth cycle. (**C**) Kaplan–Meier estimate of progression-free survival in ASPS patients with and without the expression of immunological markers; *p*-value is shown for the presence vs. absence of immunological markers. (**D**) Univariate Cox regression analysis of immunological molecules upon progression-free survival.

To investigate the composition of the TME in ASPS with a focus on immunocompetent cells, we performed Multiple Iterative Labeling by Antibody Neodeposition (MILAN) using TMAs containing 47 ASPS cases. Sequential staining against TFE3, programmed cell death 1/ligand 1(PD-1/L1), cytotoxic T-lymphocyte-associated protein 4 (CTLA-4), CD3, CD4, CD8, CD14, CD56, CD68, forkhead box protein P3 (FOXP3), and major histocompatibility complex class I/II (MHC I/II) was successfully performed in 7 cycles, and at least 45 cases were evaluable in each cycle. PD-L1 expression was seen in 10 cases (21.7%), while we observed no expression of PD-1 and only sporadic expression of CTLA-4 (4.4%, *n* = 2). As for the assessment of TILs, CD3 (T cells), CD4 (helper T cells), CD8 (cytotoxic T cells), CD14 (monocytes), CD56 (NK cells), CD68 (macrophages), and FOXP3 (regulatory T cells) were found in 17.8% (*n* = 8), 11.1% (*n* = 5), 4.4% (*n* = 2), 4.4% (*n* = 2), 0% (*n* = 0), 71.1% (*n* = 32), and 4.4% (*n* = 2) of evaluable cases, respectively. We detected MHC I and MHC II in 73.3% and 57.8% of cases, respectively, suggesting effective antigen presentation ability in ASPS. In 51.1% of cases (*n* = 23), we found co-expression of MHC I and II, whereas 22.2% (*n* = 10) of cases were double negative. We observed a higher percentage of cases with a T cell infiltration having a shorter PFS (*p* = 0.015, cut off: median PFS of 6 months) and a trend towards MHC II absence in the MHC I-negative cases; however, this correlation was not statistically significant. Figure 1A summarizes the results of MILAN per immunological parameter, with representative images shown in Figure 1B. Kaplan–Meier estimates and multiple variable Cox regression were performed to correlate the expression profiles of immunological markers with PFS, revealing that the presence of PD-L1 and FOXP3 were associated with shorter PFS and a higher risk of disease progression (Figure 1C,D). 

### 2.3. MET Status and MET Expression

The ASPS-specific marker TFE3 was detected by immunohistochemistry in 93.3% of cases (*n* = 42), of which two were negative for a *TFE3* rearrangement, as determined by FISH (i.e., MET status). These two MET-negative cases were analyzed *post hoc* with an Archer fusion panel, and in one of these MET-negative specimens, we detected a typical *ASPSCR1-TFE3* fusion, with an unusual breakpoint in exon 6 of *TFE3*. Interestingly, this patient had a remarkable response (PR) to crizotinib. No fusion was identified in the second case immunopositive for TFE3, but with a negative MET status. Next, we also evaluated MET expression using immunohistochemistry (Supplementary Material File S2) and compared MET expression with MET status and TFE3 expression (Figure 1A). We observed a consistent positivity in MET-positive ASPS, with TFE3 expression and MET expression in the majority of cases, but also discrepancies in 6 cases (2 with no *TFE3* rearrangement by FISH but positive for TFE3 and/or MET expression; 4 with positive *TFE3* rearrangement but negative for TFE3 and/or MET expression).

### 2.4. Copy Number Alteration Profile

**Figure 2:**
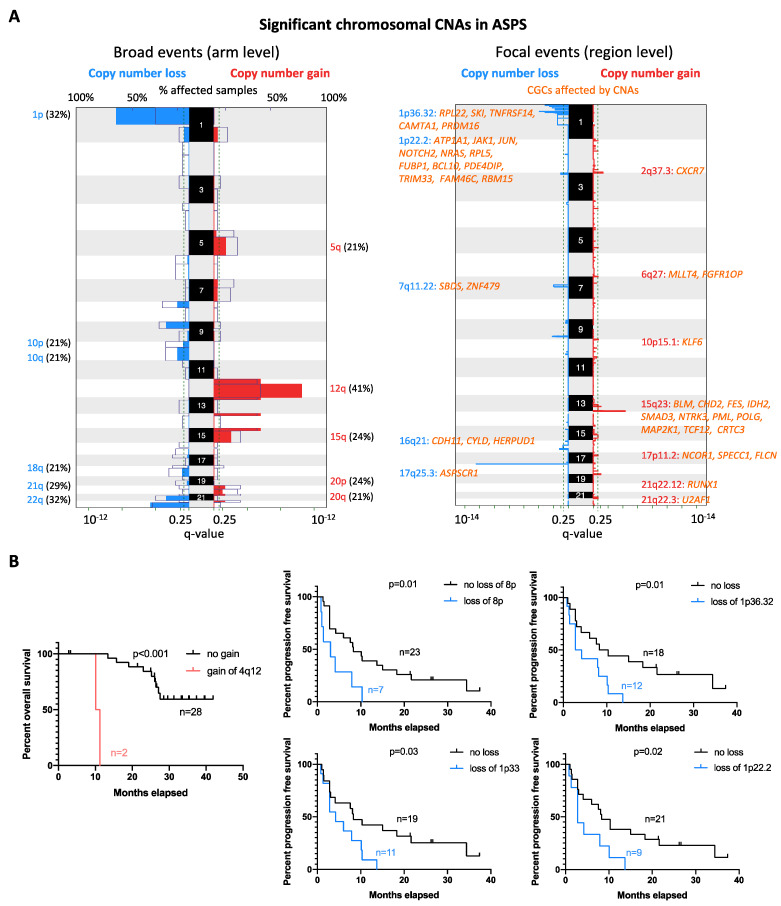
Global CNA profiles and their correlation with survival. (**A**) Recurrent alterations were identified at chromosomal arm (broad) and region levels (focal) in 24 cases. Colored peaks represent gains/losses by broad (chromosome arm) or focal (region) events; the threshold of significance is q-value < 0.25; numbers in bracket represent % of samples affected by copy number alterations. CGCs that were affected by focal CNAs are listed and coded in orange. (**B**) Kaplan–Meier estimate of overall and progression-free survival in ASPS patients with different statuses of can; *p*-value is shown for cases with the copy number change vs. no change.

Next, we performed low-coverage whole genome sequencing and Genomic Identification of Significant Targets in Cancer (GISTIC) analysis to reveal the copy number alteration (CNA) profile of ASPS in 34 assessable samples. Recurrent regions affected by broad (chromosomal arm level) and focal (region level) CNAs were detected. The most common broad CNA was a gain of chromosome 12q, observed in 14 out of 34 (41%) ASPS cases. Other broad CNA events were gains of chromosomal arm 12p (38%), 15q (24%), 20p (24%), 5q (21%), and 20q (21%), while losses mainly affected chromosome 1p (32%), 22q (32%), 9p (29%), 21q (29%), 8p (24%), 10p (21%), 10q (21%), and 18q (21%) (Figure 2A). Furthermore, focal CNAs were detected in 23 loci (15 gains and 8 deletions). The most common focal CNA was loss of 1p36.32 (44%), followed by 1p33 (38%), 17q25.3 (38%), 1p22.2 (32%), 9q33.1 (29%), 3p26.1 (24%), 7q11.22 (24%), and 16q21 (18%). Recurrent gains were observed at 15q23 (29%), 2q37.3 (26%), 13q34 (26%), 18q23 (26%), 5q31.3 (24%), 10p15.1 (21%), 6q27 (18%), 21q22.3 (18%), 2p11.2 (15%), 2p25.1 (12%), 17p11.2 (12%), 4q12 (6%), and 21q22.12 (6%). Notably, losses of 1p36.32, 1p33, and 1p22.2 co-occurred in 11 cases. In Figure 2A, the Cancer Gene Consensus related genes (CGCs) from regions most significantly affected by CNAs are listed. To determine the association between the CNA profile and clinical outcome, we correlated the recurrent CNAs with clinical features, including disease status (primary vs. metastatic), survival, and response to crizotinib. Gains of 4q12 correlated with shorter OS (*p* < 0.001), while losses of 8p (*p* = 0.01), 1p36.32 (*p* = 0.01), 1p33 (*p* = 0.03), and 1p22.2 (*p* = 0.02) were associated with shorter PFS in patients receiving crizotinib treatment (Figure 2B). No significant association with other parameters (disease status or response to crizotinib) was found. 

### 2.5. Mutational Landscape of ASPS

**Figure 3:**
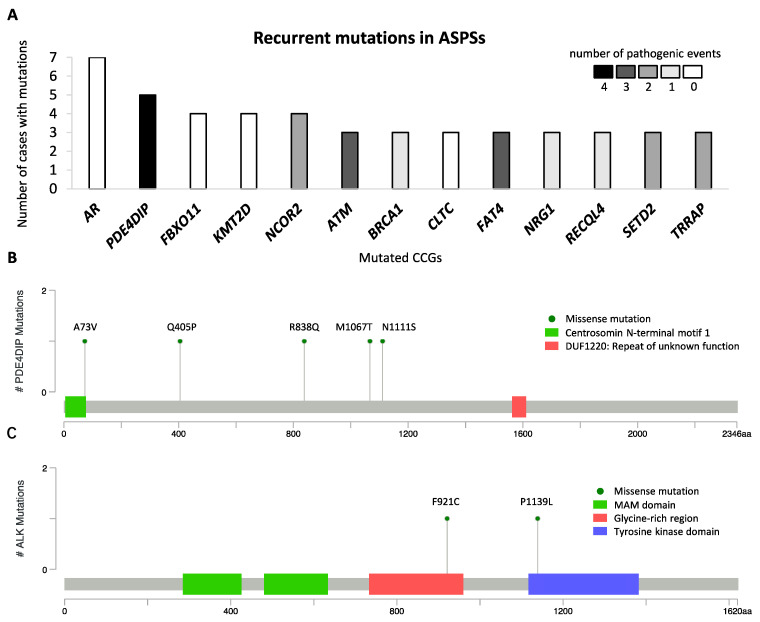
Mutational profile in alveolar soft part sarcoma, with recurrent mutations identified in 34 cases. (**A**) Cancer consensus genes altered by nonsynonymous mutations in at least 3 of 34 patients with alveolar soft part sarcoma. The *y*-axis represents the number of cases with nonsynonymous mutations, and the *x*-axis represents mutated genes. The number of predictively pathogenic events is coded in grayscale. The lollipop plots mapped the mutations in (**B**) *PDE4DIP* and (**C**) *ALK* on the linear protein sequence and their domains (colored boxes). The *Y*-axis represents the number of cases with mutations, The *x*-axis represents the amino acid sequence of mutated genes, and aa stands for amino acids.

To describe the mutational landscape in ASPS, whole-exome sequencing was performed using DNA libraries prepared for CNA analysis. A total of 8469 mutations were detected in 34 cases, with an average of 249 mutations per sample (range 174–323). Excluding synonymous mutations, we identified 4795 missense mutations, 123 nonsense mutations, and 772 insertions and deletions (indels). For further analysis, we focused on genes that were previously documented as CGCs. A total of 256 mutations that affected 175 CGCs were identified, with an average of 8 (range 2–14) per case. Of these, *AR* (androgen receptor) mutations were found in 7 out of 34 cases as the most common alteration, and 13 were found to be mutated in at least three specimens (Figure 3A). Because all 7 *AR* mutations detected were in-frame indels, occurring at a highly repetitive/polymorphic region of the *AR* domain and predicted as benign, they were not considered in the subsequent analysis. Other CGCs, mutated in at least three cases, encoded proteins involved in *PDE4DIP* (phosphodiesterase 4D interacting protein), *FBXO11*, *KMT2D*, *NCOR2*, *ATM*, *BRCA1*, *CLTC*, *FAT4*, *NRG1*, *RECQL4*, *SETD2*, and *TRRAP* (Figure 3A). Of note, all *PDE4DIP* alterations were missense mutations, randomly occurring at different locations in 5 cases, and 4 of which were bioinformatically predicted as pathogenic events (Figure 3B). Furthermore, we detected two *ALK* mutations (p.F921C and p.P1139L) in MET-positive ASPS tumors from patients who achieved PR or SD as the best response to crizotinib. The *ALK* p.F921C mutation was reported in squamous cell carcinoma and adenocarcinoma and were predicted as pathogenic, while the *ALK* p.P1139L mutation was a novel variant with uncertain clinical significance [14]. These *ALK* substitutions were located in the glycine-rich region and tyrosine kinase domain, respectively (Figure 3C). To date, the function of the glycine-rich region within human *ALK* is still unclear, while the tyrosine kinase domain has catalytic activity [15]. Among genes mutated in more than one case, we used Drug Gene Interaction Database (DGIdb) to identify 20 potentially druggable targets, including *AR*, *NCOR2*, *ATM*, *BRCA1*, *FAT4*, *NRG1*, *TRRAP*, *ALK*, *COL1A1*, *CREBBP*, *EGFR*, *EP300*, *FAT1*, *FLT4*, *LIFR*, *NOTCH1*, *NR4A3*, *PTPRB*, *RANBP2*, and *SMO*.

### 2.6. Molecular Landscape of ASPS

**Figure 4:**
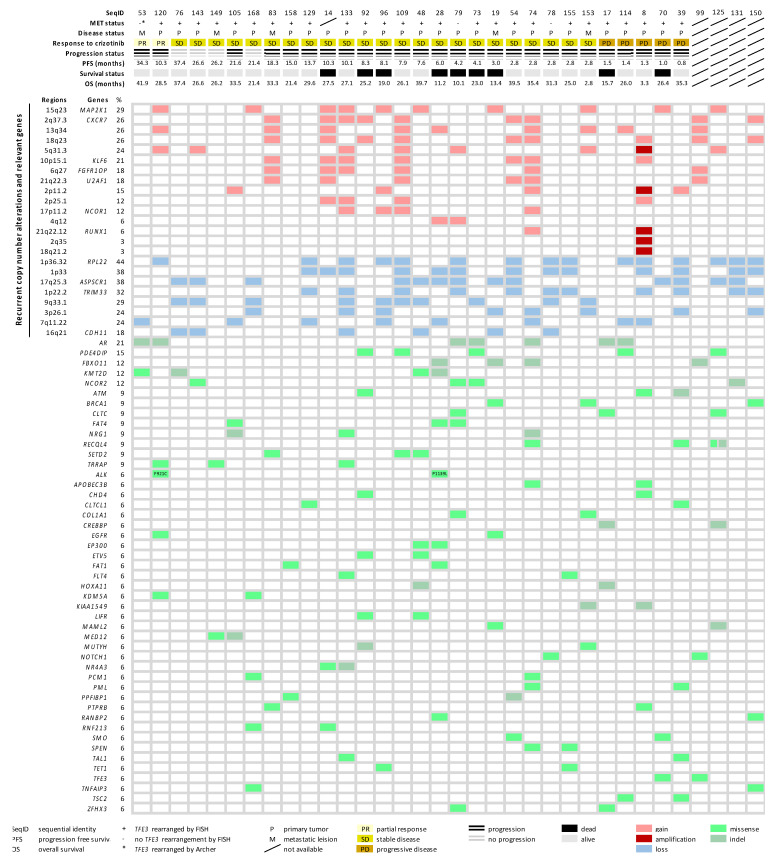
A summarized heatmap for the gene alteration landscape and trial-related clinical data. Clinical data for each case was listed on the top in the order of response to crizotinib. Types of alterations that affected genomic regions, as well as genes (cancer consensus gene associated genes), were sorted according to frequency. Rows represent individual regions and genes, while columns represent individual cases. Clinical features and types of alterations and are coded in different colors (bottom).

**Figure 5:**
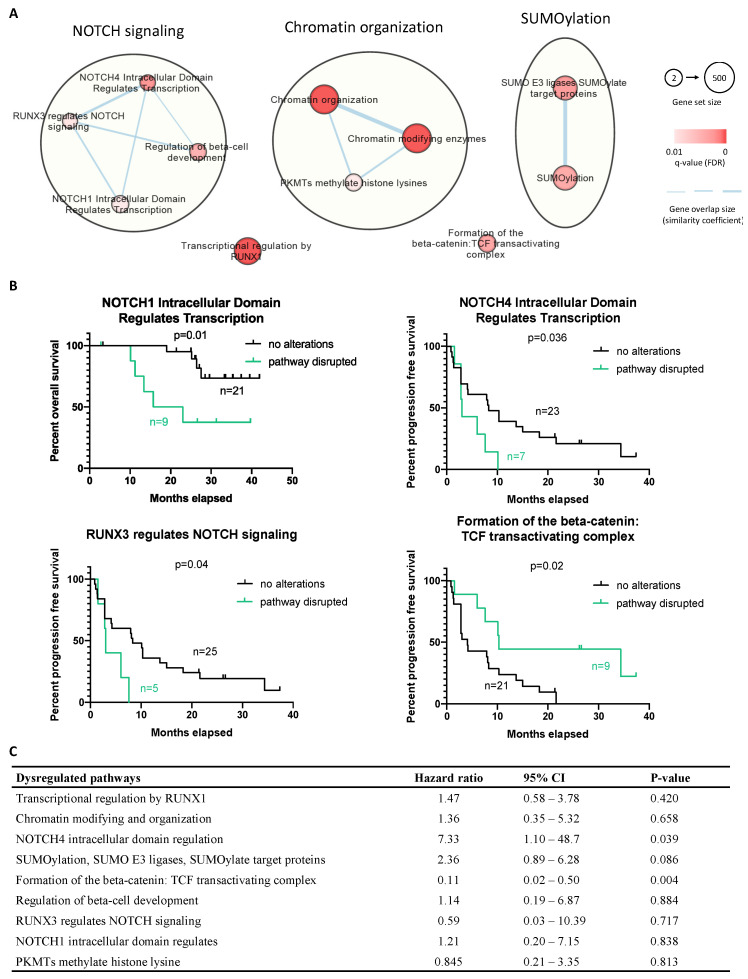
Integrative pathway enrichment analysis using the mutated gene list in the CGC set and the association between altered pathways and patients’ survival. (**A**) Dysregulated pathways were mainly related to NOTCH signaling, chromatin organization, and SUMOylation damage. Red-coded nodes represented as the significantly dysregulated pathways, and the significance was marked by color intensity. The size of the node represents gene set size (number of genes documented in each pathway). The edges (gene overlap size), represented as the associations between pathways and the thickness, were used to present the associated level. (**B**) Progression-free survival for cases with and without dysregulated pathways. (**C**) Univariate Cox regression analysis of dysregulated pathways upon progression-free survival; *p*-value < 0.05 is considered significant.

Next, we combined the molecular findings from the CNA and mutational analysis, aiming to explore the disease biology beyond *TFE3* rearrangement. The molecular landscape is summarized in Figure 4. Furthermore, by pathway enrichment analysis, we allocated the mutated CGCs to predefined pathways (Reactome), aiming to identify significant dysregulations at the pathway level. We identified 11 disrupted pathways, with 9 of them being related to NOTCH signaling, chromatin organization, and SUMOylation (Figure 5A). The overrepresented terms of disrupted pathways are listed in Supplementary Material File S3. To identify potential predictive factors, we correlated the altered pathways with PFS. Figure 5B shows that three altered pathways related to NOTCH signaling were associated with shorter PFS, while longer PFS was seen in cases with dysregulation in the beta-catenin/T-cell factor (TCF) complex. Furthermore, univariate Cox regression was performed, demonstrating a higher risk of progression in cases of NOTCH4 intracellular domain dysregulation, while the beta-catenin/TCF complex pathway was associated with a lower risk of progression (Figure 5C).

## 3. Discussion

ASPS is an ultra-rare subtype of STS that is characterized by the *ASPSCR1-TFE3* fusion, resulting in MET overexpression. In the prospective phase II trial EORTC 90,101 “CREATE”, the activity of the MET inhibitor crizotinib was assessed in patients with this rare disease. Clinical results suggested the presence of factors other than *TFE3* rearrangement that might predict treatment outcome [6]. Moreover, recent studies indicated that ASPS is likely sensitive to ICIs, but the reasons for this are still unclear [10,11]. Using one of the largest collections of biological material from ASPS (*n* = 47) originating from the CREATE trial, we were able to characterize the composition of the TME and genomic alterations in the tumor tissue and further correlate these findings with the patient outcome on crizotinib treatment, aiming to identify potential biomarkers and additional therapeutic targets. 

Since PD-L1 and immunocompetent cells in the TME have been suggested as potential predictors for patient outcomes on both tyrosine kinase inhibition (TKI) treatment and immunotherapy in various cancers [9,12,16,17,18], it was intriguing to see whether such TME signatures could have an impact on the natural course and crizotinib treatment of ASPS. We therefore evaluated the expression of immune checkpoints and the presence of specific TIL populations in a TMA. PD-1 was not expressed, and CTLA-4 was found in only 4% of cases. PD-L1 presented in 22% of cases. PD-L1 has also been reported as a predictor for a poor prognosis in STSs, which was attributed to a favorable condition for immune exhaustion and tumor evasion [19]. In the present study, we found a correlation between PD-L1 expression and worse PFS and a higher risk for disease progression, displaying potential predictive and prognostic value of this immune checkpoint for the clinical outcome on crizotinib treatment. Since previous reports demonstrated that the efficacy of EGFR inhibitors differed based on TME composition in NSCLC (i.e., a subset of samples with high PD-L1 and CD8 expression poorly benefitted from targeted treatment) [9], we also correlated the immunological profiles in ASPS with clinical data related to crizotinib treatment. The presence of FOXP3-positive regulatory T cells was associated with worse patient outcome, but due to the unbalanced sample sizes in FOXP3-positive (*n* = 2) and FOXP3-negative (*n* = 41) groups, we cannot overestimate the predictive power. Apart from the sparse presence of TILs (0–8%), macrophages (CD68-positive cells) were observed in 71% of cases, which was in line with a recent study showing that CD68-positive macrophages outnumbered TILs in various sarcomas [18]. Such dominance of tumor-associated macrophages over TILs in TME suggested a treatment approach targeting tumor-associated macrophages that may improve the outcome of immunotherapy in ASPS patients [20]. On the other hand, 22% of cases were positive for PD-L1, but no PD-1 expression was seen in the analyzed study, suggesting a potential role of anti-PD-L1 treatment in ASPS. Our investigation of immune checkpoints somehow matched the results of recent trials that tested various ICIs in ASPS. In an ongoing phase II trial testing atezolizumab (anti-PD-L1), 16 out of 43 cases achieved at least PR [10]. Among 8 cases with evaluable biopsy pairs, in all cases, both baseline and post-treatment specimens demonstrated PD-L1 expression. Limited cases treated with nivolumab (anti-PD-1) achieved CR or PR, as seen in a case report and a phase II “OSARC” trial (NCCH1510) [21,22]. In summary, our study represented a comprehensive profile and the impacts of immunological components on crizotinib treatment in the present ASPS cohort. A better understanding of the TME signature could contribute to improving the therapeutic strategies of TKIs, ICIs, or even their combinations in ASPS patients.

Interestingly, a MET-negative case that had a remarkable response to crizotinib was positive for TFE3 expression. We therefore performed additional Archer analysis and identified an *ASPSCR1-TFE3* fusion with an unusual *TFE3* breakpoint (exon 6). Although *ASPSR1* is more often fused to *TFE3* at either exon 3 or 4, *ASPSCR1-TFE3* can also involve the fusion of exons 1–7 of *ASPSCR1* to exon 5 or 6 of *TFE3* [23,24]. Our result shows that the FISH probes (CHRX: 48010297-48212489, CHRX: 51569622–51753153) that we used failed to flank the *TFE3*/*Xp11.2* gene in this case, highlighting the importance of an additional methodology to identify *TFE3*-rearranged ASPS. In another MET-negative case with TFE3 expression, no fusion was identified, suggesting an alternative mechanism inducing TFE3 expression in the absence of *TFE3* rearrangement. For instance, cellular stressors such as DNA damage and oxidative stress have been implicated in activating TFE3 [25]. Moreover, we observed that three cases with TFE3 expression and/or *TFE3* rearrangement were negative for MET expression. Likewise, two cases without TFE3 expression or *TFE3* rearrangement expressed MET. These suggested the presence of other factors that may result in MET down-/upregulation. For instance, DAXX (a highly conserved nuclear protein that represses the activity of transcriptional factors) binding to the *MET* promoter can result in the transcriptional repression of *MET* [26], while AP1-enhancing transcription and eIF-mediated translation were considered as alternative mechanisms resulting in MET upregulation [27]. Nevertheless, these counterintuitive observations require further investigation, and the sarcomagenesis of TFE3-negative ASPS remains to be explored.

By low-coverage whole-genome sequencing, we aimed to identify significant CNAs in ASPS. The most common CNAs observed were gains of chromosome 12q (41%) and losses of 1p36.32 (44%) as broad and focal alterations, respectively. Similar to our observation, the gain of the 12q amplicon was previously found in individual ASPS cases [28,29], and the putatively amplified oncogenes on chromosome 12q (e.g., *CDK4* and *MDM2*) were frequently seen in patients with soft tissue and bone tumors [30,31]. This may imply that genes affected by these disease-associated CNAs may have oncogenic roles in ASPS sarcomagenesis, suggesting the potential utility of CDK4/MDM2 inhibitors for the treatment of this disease. Other genes located on 12q and implicated in STSs could also be of interest. For instance, *GLI1* amplification may serve as an alternative mechanism of oncogenic activation akin to *GLI1* fusions, which was reported to contribute to the development of STS [32]. In a correlative analysis, we observed that loss of chromosome 8p and, regions of 1p36.32, 1p33, 1p22.2 were associated with shorter PFS in patients receiving crizotinib. Among these altered regions, chromosome 1p36 was found deleted in various human cancers and reported as a predictor for disease progression of neuroblastoma [33,34]. Within a region of 1p36.32, adherens junction-associated protein-1 (*AJAP1*) encoding an integral membrane protein has been considered as a tumor suppressor. The loss of AJAP1 expression (caused by deletion or methylation) was associated with poor clinical outcome in patients with malignant gliomas [35]. Therefore, previously described alterations in ASPS could be worthy of further investigation, suggesting the potentially predictive values for the clinical outcomes of ASPS patients on crizotinib treatment.

Next, we investigated the ASPS mutational profile using WES and focused on recurrent mutations identified in our cohort. The most frequently mutated gene was *PDE4DIP*, a gene transcribing myomegalin that is important for the engagement of 4D phosphodiesterase to the Golgi complex [36]. Mutations in this gene have been reported to possibly cause myeloproliferative disorders associated with eosinophilia, but it is also found in various cancers including STS (e.g., endometrial stromal sarcoma) [37,38]. This is the first time that *PDE4DIP* was identified as a putative susceptibility gene in ASPS. A recent study has also characterized the molecular alterations in ASPS, derived from the American Association for Cancer Research GENIE database, revealing 40 cancer-associated mutations detected in 20 ASPS tumors [39]. Of these, mutations in *KMT2D* (lysine methyltransferase 2D), *ATM* (ATM serine/threonine kinase), and *RECQL4* (ATP-dependent DNA helicase Q4) were also found in our cohort of 34 cases. The proteins encoded by *KMT2D*, *ATM*, and *RECQL4* are implicated in epigenetic modification, DNA damage repair, and genome caretaking, respectively [40,41,42]. This observation suggested that these alterations and affected pathways were likely involved in ASPS biology. Furthermore, we identified two *ALK* mutations located at the glycine-rich region (p.F921C) and the tyrosine kinase domain (p.P1139L) in two TFE3-positive cases that achieved a PR and SD on crizotinib treatment, respectively. These alterations were either predictively pathogenic or located in the catalytic region, suggesting a potential impact of *ALK* mutations on the sensitivity to crizotinib, as previously described in various tumor types (e.g., NSCLC, anaplastic large-cell lymphoma and inflammatory myofibroblastic tumor) [43,44,45]. However, we were not able to determine their association response to crizotinib due to the inclusive contribution of the TFE3-MET axis, as well as the lack of functional information on newly discovered variants. The coexistence of *ALK*, *MET*, and other alterations has been observed in a series of cases with lung cancer [46] and is reported here in MET-positive ASPS tumors with *ALK* mutations.

We combined genomic alterations identified in this study, revealing the molecular heterogeneity in ASPS. To further investigate the overrepresented dysregulations in molecular pathways, we further analyzed the mutational dataset to identify pre-defined pathways enriched with mutated CGCs. Our findings showed that disease-associated alterations might be actively involved in NOTCH signaling, chromatin organization, and SUMOylation (small ubiquitin-like modifier). Notably, SUMOylation has already been related to the functionality of TFE3, which possesses a SUMOylation site and can be post-transcriptionally modified to regulate the transcriptional activity [47]. The mutations affecting SUMOylation could therefore impact on the transcriptional activity of TFE3 and could have a functional consequence on MET expression and the response to crizotinib, although this hypothesis requires further investigation. More interestingly, we observed that NOTCH signaling (a cell-surface receptor transducing signal to promote cancer development) was associated with shorter survival in the analyzed ASPS. In more detail, dysregulated NOTCH4 signaling was more often seen in patients with shorter PFS and a higher risk for disease progression, suggesting its predictive value, but also potential NOTCH-targeting therapeutic strategies, as described in the literature [48]. On the other hand, we uncovered that dysregulation in the beta-catenin-TCF transactivating complex was linked with better treatment outcomes. Possibly, this transcription activator was altered and became disabled to induce its targeted genes, including MYC, cyclin D1, and matrix metalloproteinase-7 [49]. It was also reported that the knockdown of beta-catenin can restore the response to crizotinib in neuroblastoma [50], which may support the observed positive effect of beta-catenin deficiency in our cohort and imply a strategy of combining inhibitors against beta-catenin and MET/ALK.

This study has some limitations. First of all, because of the unavailability of matching germline samples, we used computational strategies with restricted criteria to exclude common single nucleotide polymorphism. However, it is still possible to have a small proportion of germline polymorphisms included in our dataset. Secondly, although the public databases (e.g., Catalogue of Somatic Mutations in Cancer databases (COSMIC) and Reactome) we used to study genomic alterations are extensive and well-annotated, novel alterations and their functionality are still being documented over time. Emerging genomic data and new annotations should be implemented for better clinical interpretation, and further investigation is still required to validate the observations in this study. Furthermore, we only focused on cancer-related genes (CGCs), which might overlook rare or uninterpreted alterations in the process of variant identification. Nevertheless, to the best of our knowledge, this study is one of the largest ASPS study cohorts, in which we present a comprehensive molecular analysis of this rare disease.

## 4. Material and Methods

### 4.1. Characterization of Immunological Components in the Tumor Microenvironment

A total of 47 archived formalin-fixed, paraffin-embedded (FFPE) tissue samples from patients with ASPS were collected in the context of the clinical trial and were used for the construction of an ASPS-specific TMA with 1.5 mm triplicate cores per case [51]. To detect immunological components in the TME, we applied the MILAN technique, a multiplex immunoassay involving repeated cycles of indirect immunofluorescence, image acquisition, and antibody removal [52,53]. We optimized the procedure for TMA sections and defined the sequence of cycles as previously described (Supplementary Material File S1) [51]. Immunofluorescence staining was scored by the first author of this manuscript and was assessed as a categorical variable based on the percentage of cells expressing targeted molecules (i.e., 0: negative, 1: 1–10% of stained cells, 2: 10–30% of stained cells, or 3: >30% of stained cells). Cores with over 80% of tissue loss were considered unevaluable. For cases with more than one evaluable core, the mean of the scoring was recorded as the final result. The targeted molecules and corresponding evaluation criteria were the following: membranous expression for the PD-1, PD-L1, CTLA-4, CD3, CD4, CD8, CD14, CD56, CD68, and MHC I/II. Nuclear expression for TFE3 (ASPS-specific molecule), and both cytoplasmic and nuclear expression for FOXP3.

### 4.2. MET Status and MET Expression

In the CREATE trial, MET status was indirectly assessed centrally by FISH, as described in the study protocol (accessed on 17 December 2021 http://www.eortc.be/services/doc/protocols/90101v10.0.pdf). A tumor was considered MET-positive if it showed *TFE3* rearrangement in at least 15% of cells by FISH (home-made break-apart TFE3 probe set RP11-344N17 and RP11-552J9) [6]. We also performed a *post hoc* targeted next-generation sequencing-based Archer FusionPlex CTL Panel (Archer, Boulder, USA), to evaluate the presence of specific fusions in MET-negative ASPS specimens. MET expression was evaluated using immunohistochemistry with anti-MET monoclonal antibody (D1C2, Cell Signaling Technology, Danvers, MA, USA).

### 4.3. Low-Coverage Whole-Genome Sequencing

We isolated DNA from FFPE tumor samples, and 34 out of 47 samples were available (other samples were either limited or of low quality), with quality that was acceptable for DNA libraries preparation. Illumina^®^ HiSeq4000 (Illumina, San Diego, CA, USA) was used for sequencing at low coverage (± 0.1×). Raw sequencing reads (50 bp) were mapped to the human reference genome (GRCh37/hg19 version) using Burrows-Wheeler Aligner (BWA v0.5.8a, Massachusetts Institute of Technology, USA) and sorted with SAMtools (v0.1.19, Massachusetts Institute of Technology, Cambridge, MA, USA). Picard tools were used to remove duplicates. QDNASeq and ASCAT were used to count and segment aligned reads in bins of 50k [54,55]. The GISTIC algorithm (Broad Institute, Cambridge, MA, USA) was used to identify the most frequent and significant chromosomal alterations. A region was considered deleted if the log value was <−0.1, while it was amplified if the log value was >0.1. The Benjamini–Hochberg method was used to correct for multiple testing [56], and significant CNAs were selected, with a cut-off q-value < 0.25. We defined CNA as a broad (arm level) event if alterations spanned > 75% of a chromosomal arm. Alterations spanning < 75% of a chromosomal arm were considered as focal CNAs (region level).

### 4.4. Whole Exome Sequencing

Libraries prepared for low-coverage whole-genome sequencing were enriched for exomic sequences using the SeqCapV3 exome enrichment kit (Roche, Switzerland) following the manufacturer’s instructions. They were sequenced on HiSeq4000 using a V3 flow cell, resulting in 2 × 150 bp paired-end reads that were further mapped and sorted as described above. Duplicate reads were removed using Picard tools (Broad Institute, Cambridge, MA, USA), followed by base recalibration, local realignment, and single nucleotide variant calling performed with the Genome Analysis Tool Kit (GATK, Broad Institute, Cambridge, MA, USA). Low-quality mutations were excluded if the coverage for calling substitutions, insertions, and deletions (indels) was less than 10×. Furthermore, Dindel was used for calling insertions and deletions (indels), with criteria of a quality score of more than 50 and at least 10x coverage. Since no germline samples were available, a strict filtering strategy was applied based on publicly available databases to exclude the common single nucleotide polymorphisms. Mutations occurring in large databases (ESP, 1 kg, ExAC) with an allelic frequency > 0.001, as well as mutations occurring in smaller, appropriate databases (bitsTrio, inhouseDB, cg69, GoNL, Illumina, San Diego, CA, USA) were removed if they occurred in more than one individual. The CGC set developed by the COSMIC v89 (Wellcome Trust Sanger Institute, Hinxton, UK) was then applied to include genes that have been implied in cancer for further analysis [57]. The visualization of mutations was performed using MutationMapper (cBioPortal, Memorial Sloan-Kettering Cancer Center, New York, USA) [58]. The bioinformatic prediction of the pathogenicity of variants was carried out with the human genomic variant search engine VarSome (Saphetor SA, Lausanne, Switzerland) [59]. To assess the clinical application of mutated genes, we used the DGIdb (Washington University School of Medicine, USA) to predict the potential druggability [60].

### 4.5. Pathway Enrichment Analysis

To identify affected pathways that are mutation-mediated in ASPS, pathway enrichment analysis was performed using g:Profiler (University of Tartu, Tartu, Estonia), a tool integrating bioinformatic and statistic approaches to call pathways whose genes are significantly enriched or overrepresented in a list of genes, as compared to all genes in the genome [61]. We allocated the mutated CGCs to predefined pathways based on Reactome (ELIXIR, Cambridge, UK), one of the most common molecular-pathway databases [62]. Pathways with a minimum size of 5 genes per set were considered for enrichment analysis. The significance was calculated based on Fisher’s exact test and multiple-test correction. Pathways with a q-value less than 0.01 were considered significant. EnrichmentMAP, AutoAnnotate, and the Markov Cluster Algorithm (Bader Lab, University of Toronto, Toronto, Canada) were used for visualization and annotation in a Java-based computational environment, Cytoscape v3.7.2 (Institute of Systems Biology, Seattle, WA, USA) [63,64]. 

### 4.6. Clinical Outcome and Statistical Analysis

The response to crizotinib in the clinical trial was evaluated using Response Evaluation Criteria in Solid Tumors (RECIST v1.1), as previously reported [6]. In the present exploratory translational study, 43 out of 47 patients were eligible for primary and secondary endpoints. The primary endpoint was an objective response based on RECIST v1.1. Secondary endpoints included PFS and OS, as described in the original publication [6]. For the current analysis, Fisher’s exact test was used to test if the defined-response groups were significantly enriched for certain alterations. The Kaplan–Meier estimate with a log-rank test was used to assess the correlation between molecular findings and survival. Cox regression analysis was also used for a multiple variable analysis of survival. For correlations between survival and the expression of immunological components of the microenvironment, Kaplan–Meier estimates were calculated for cases without (0: negative) and with the expression of selected markers (1: 1–10% of stained cells, 2: 10–30% of stained cells, or 3: >30% of stained cells). Cox regression was tested in categories (0: negative, 1: 1–10% of stained cells, 2: 10–30% of stained cells, or 3: >30% of stained cells) based on the marker expression level, evaluated by MILAN. Statistical analysis was performed using GraphPad Prism v7 and SPSS v27; *p* values < 0.05 were considered significant. 

## 5. Conclusions

We characterized the composition of the TME and genomic alterations in ASPS, revealing molecular heterogeneity. The correlation of molecular findings with patient outcome revealed potential biomarkers with predictive value for crizotinib treatment. Our study provides insights into the biology of this complex and clinically challenging ultra-rare malignancy, which may pave the way for the development of better therapeutic strategies for ASPS patients.

## Figures and Tables

**Table 1:** Patient characteristics and availability of biological material.

Study SeqID	Gender/Age	Tissue Source/ Type of Lesion	MET Status (EORTC 90,101 Protocol)		Best Response (RECIST)	Progression Status on Crizotinib	PFS (Months)	Survival Status	OS (Months)	Exploratory Study
			Status	% Cells Positive for FISH						
4	M/51	Primary	MET +	nd	PD	Progression	1.5	Death	14.7	TMA+Sequencing
7	M/25	Metastatic	MET +	nd	SD	Progression	2.5	Death	39.4	TMA
8	F/28	Primary	MET +	nd	PD	Progression	1.3	Alive	3.3	TMA
14	M/35	Primary	nd	nd	SD	Progression	10.3	Death	27.5	TMA+Sequencing
16	M/30	Metastatic	MET +	60	SD	Progression	12.8	Alive	39.0	TMA
17	F/21	Primary	MET +	85	PD	Progression	1.5	Death	15.7	TMA+Sequencing
19	M/23	Metastatic	MET +	91	SD	Progression	3.0	Death	13.4	TMA+Sequencing
24	F/31	Primary	MET +	80	SD	Progression	4.2	Death	40.1	TMA
28	M/33	Primary	MET +	61	SD	Progression	6.0	Death	11.2	TMA+Sequencing
39	M/51	Primary	MET +	nd	PD	Progression	0.8	Alive	35.3	TMA+Sequencing
48	M/30	Primary	MET +	75	SD	Progression	7.6	Alive	39.7	TMA+Sequencing
53	M/69	Metastatic	MET − *	0	PR	Progression	34.4	Alive	41.9	TMA+Sequencing
54	M/54	Primary	MET +	61	SD	Progression	2.8	Alive	39.5	TMA+Sequencing
61	M/43	Primary	MET +	31	SD	Progression	4.2	Alive	39.1	TMA
65	F/42	Primary	MET +	61	SD	Progression	38.8	Alive	41.9	TMA
67	M/34	Primary	MET +	31	SD	No progression	33.5	Alive	33.5	TMA
70	M/33	Primary	MET +	55	PD	Progression	1.0	Death	26.4	TMA+Sequencing
73	F/18	Primary	MET +	67	SD	Progression	4.1	Death	23.0	TMA+Sequencing
74	F/20	Primary	MET +	21	SD	Progression	2.8	Alive	35.4	TMA+Sequencing
76	M/24	Primary	MET +	76	SD	No progression	37.4	Alive	37.4	TMA+Sequencing
77	F/40	Primary	MET +	69	SD	Progression	11.6	Death	20.3	TMA
78	M/37	Primary	MET −	0	SD	Progression	2.8	Alive	31.3	TMA+Sequencing
79	M/45	Primary	MET −	0	SD	Progression	4.2	Death	10.1	TMA+Sequencing
83	F/19	Metastatic	MET +	74	SD	Progression	18.3	Alive	33.3	TMA+Sequencing
89	F/33	Primary	MET +	43	SD	Progression	5.7	Alive	24.6	TMA
90	F/22	Metastatic	MET +	31	SD	Progression	4.1	Alive	33.3	TMA
92	M/29	Primary	MET +	24	SD	Progression	8.3	Death	25.2	TMA+Sequencing
93	M/24	Metastatic	MET +	43	SD	Progression	4.2	Death	10.8	TMA
96	F/28	Primary	MET +	82	SD	Progression	8.1	Death	19.0	TMA+Sequencing
97	F/37	Primary	MET +	81	SD	Progression	1.8	Alive	30.0	TMA
98	M/33	Primary	MET +	51	SD	Progression	15.2	Alive	34.6	TMA
99	M/17	Primary	MET +	67	nd	nd	nd	Alive	nd	TMA+Sequencing
105	M/37	Primary	MET +	60	SD	Progression	21.6	Alive	33.5	TMA+Sequencing
109	M/39	Primary	MET +	80	SD	Progression	7.9	Alive	26.1	TMA+Sequencing
114	F/28	Primary	MET +	91	PD	Progression	1.4	Alive	26.0	TMA+Sequencing
120	M/31	Primary	MET +	37	PR	Progression	10.3	Alive	28.5	TMA+Sequencing
125	F/32	Metastatic	MET +	26	nd	nd	nd	Alive	nd	TMA+Sequencing
129	F/16	Primary	MET +	65	SD	Progression	13.7	Alive	29.6	TMA+Sequencing
131	M/33	Primary	MET +	47	nd	nd	nd	Alive	nd	TMA+Sequencing
133	F/45	Primary	MET +	49	SD	Progression	10.1	Alive	27.1	TMA+Sequencing
143	F/33	Primary	MET +	36	SD	No progression	26.6	Alive	26.6	TMA+Sequencing
149	F/18	Metastatic	MET +	40	SD	No progression	26.2	Alive	26.2	TMA+Sequencing
150	M/25	Primary	MET +	19	nd	nd	nd	Alive	nd	TMA+Sequencing
153	M/24	Metastatic	MET +	35	SD	Progression	2.8	Alive	2.8	TMA+Sequencing
155	F/26	Primary	MET +	46	SD	Progression	2.8	Alive	25.0	TMA+Sequencing
158	F/30	Primary	MET +	65	SD	Progression	15.0	Alive	21.4	TMA+Sequencing
168	F/34	Primary	MET +	71	SD	No progression	21.4	Alive	21.4	TMA+Sequencing

+: positive, −: negative, *: *TFE3* rearrangement positive by Archer Analysis, F: female, FISH: fluorescent in situ hybridization, M: male, nd: no data, OS: overall survival, PD: progressive disease, PFS: progression-free survival, PR: partial response, RECIST: Response Evaluation Criteria in Solid Tumors, SD: stable disease, SeqID: sequential identity, TMA: tissue microarray; data cut-off date: 8 May 2020.

## Data Availability

All data generated or analyzed during this study are included in this published article, and the DNA sequencing data used and/or analyzed during the current study can be available via reasonable request.

## Supplementary Materials

The following supporting information can be downloaded at: https://www.mdpi.com/article/10.3390/ijms23105689/s1.

## Abbreviations

ASPS	alveolar soft part sarcoma
CNA	copy number alteration
STS	Soft tissue sarcoma
ASPSCR1-TFE3	alveolar soft part sarcoma critical region 1-transcription factor E3
EORTC	European Organization for Research and Treatment of Cancer
FISH	fluorescence in situ hybridization
NSCLC	non-small cell lung cancer
PI3K	phosphoinositide 3-kinase
PD-L1	programmed cell death protein ligand 1
TIL	tumor-infiltrating lymphocyte
EGFR	epidermal growth factor receptor
TME	tumor microenvironment
FFPE	formalin-fixed, paraffin-embedded
TMA	tissue microarray
MILAN	Multiple Iteractive Labeling by Antibody Neodeposition
CTLA-4	cytotoxic T-lymphocyte-associated protein 4
PD-1	programmed cell death protein 1
MHC	major histocompatibility complex class
FOXP3	forkhead box protein P3
GISTIC	Genomic Identification of Significant Targets in Cancer
CGC	Cancer Gene Consensus
COSMIC	Catalogue of Somatic Mutations in Cancer databases
RECIST	Response Evaluation Criteria in Solid Tumors
PFS	progression-free
OS	overall survival
PR	partial response
SD	stable disease
PD	progressive disease
DAPI	4′,6-diamidino-2-phenylindole
TCF	T-cell factor
VSC	Flemish Supercomputer Center
FWO	Research Foundation—Flanders

## References

[1] de Pinieux G., Karanian M., Loarer F.L., Guellec S.L., Chabaud S., Terrier P., Bouvier C., Batistella M., Neuville A., Robin Y.-M. (2021). Nationwide Incidence of Sarcomas and Connective Tissue Tumors of Intermediate Malignancy over Four Years Using an Expert Pathology Review Network. PLoS ONE.

[2] Folpe A.L., Deyrup A.T. (2006). Alveolar Soft-part Sarcoma: A Review and Update. J. Clin. Pathol..

[3] Paoluzzi L., Maki R.G. (2019). Diagnosis, Prognosis, and Treatment of Alveolar Soft-Part Sarcoma: A Review. JAMA Oncol..

[4] WHO Classification of Tumours Editorial Board (2020). World Health Organization Classification of Tumours.

[5] Tsuda M., Davis I.J., Argani P., Shukla N., McGill G.G., Nagai M., Saito T., Laé M., Fisher D.E., Ladanyi M. (2007). TFE3 Fusions Activate MET Signaling by Transcriptional Up-Regulation, Defining Another Class of Tumors as Candidates for Therapeutic MET Inhibition. Cancer Res..

[6] Schöffski P., Wozniak A., Kasper B., Aamdal S., Leahy M.G., Rutkowski P., Bauer S., Gelderblom H., Italiano A., Lindner L.H. (2018). Activity and Safety of Crizotinib in Patients with Alveolar Soft Part Sarcoma with Rearrangement of TFE3: European Organization for Research and Treatment of Cancer (EORTC) Phase II Trial 90101 ‘CREATE’. Ann. Oncol.

[7] Drilon A., Clark J.W., Weiss J., Ou S.-H.I., Camidge D.R., Solomon B.J., Otterson G.A., Villaruz L.C., Riely G.J., Heist R.S. (2020). Antitumor Activity of Crizotinib in Lung Cancers Harboring a MET Exon 14 Alteration. Nat. Med..

[8] Jamme P., Fernandes M., Copin M.-C., Descarpentries C., Escande F., Morabito A., Grégoire V., Jamme M., Baldacci S., Tulasne D. (2020). Alterations in the PI3K Pathway Drive Resistance to MET Inhibitors in NSCLC Harboring MET Exon 14 Skipping Mutations. J. Thorac. Oncol..

[9] Matsumoto Y., Sawa K., Fukui M., Oyanagi J., Izumi M., Ogawa K., Suzumura T., Watanabe T., Kaneda H., Mitsuoka S. (2019). Impact of Tumor Microenvironment on the Efficacy of Epidermal Growth Factor Receptor-Tyrosine Kinase Inhibitors in Patients with EGFR-Mutant Non-Small Cell Lung Cancer. Cancer Sci..

[10] Naqash A.R., O’Sullivan Coyne G.H., Moore N., Sharon E., Takebe N., Fino K.K., Ferry-Galow K.V., Hu J.S., Van Tine B.A., Burgess M.A. (2021). Phase II Study of Atezolizumab in Advanced Alveolar Soft Part Sarcoma (ASPS). J. Clin. Oncol..

[11] Lewin J., Davidson S., Anderson N.D., Lau B.Y., Kelly J., Tabori U., Salah S., Butler M.O., Aung K.L., Shlien A. (2018). Response to Immune Checkpoint Inhibition in Two Patients with Alveolar Soft-Part Sarcoma. Cancer Immunol. Res..

[12] Gibney G.T., Weiner L.M., Atkins M.B. (2016). Predictive Biomarkers for Checkpoint Inhibitor-Based Immunotherapy. Lancet Oncol..

[13] Koirala P., Roth M.E., Gill J., Piperdi S., Chinai J.M., Geller D.S., Hoang B.H., Park A., Fremed M.A., Zang X. (2016). Immune Infiltration and PD-L1 Expression in the Tumor Microenvironment Are Prognostic in Osteosarcoma. Sci. Rep..

[14] Tate J.G., Bamford S., Jubb H.C., Sondka Z., Beare D.M., Bindal N., Boutselakis H., Cole C.G., Creatore C., Dawson E. (2019). COSMIC: The Catalogue Of Somatic Mutations In Cancer. Nucleic Acids Res..

[15] Huang H. (2018). Anaplastic Lymphoma Kinase (ALK) Receptor Tyrosine Kinase: A Catalytic Receptor with Many Faces. Int. J. Mol. Sci..

[16] Wei Z.-W., Wu J., Huang W.-B., Li J., Lu X.-F., Yuan Y.-J., Xiong W.-J., Zhang X.-H., Wang W., He Y.-L. (2020). Immune-Infiltration Based Signature as a Novel Prognostic Biomarker in Gastrointestinal Stromal Tumour. EBioMedicine.

[17] Lin Z., Liu L., Xia Y., Chen X., Xiong Y., Qu Y., Wang J., Bai Q., Guo J., Xu J. (2018). Tumor Infiltrating CD19+ B Lymphocytes Predict Prognostic and Therapeutic Benefits in Metastatic Renal Cell Carcinoma Patients Treated with Tyrosine Kinase Inhibitors. Oncoimmunology.

[18] Dancsok A.R., Gao D., Lee A.F., Steigen S.E., Blay J.-Y., Thomas D.M., Maki R.G., Nielsen T.O., Demicco E.G. (2020). Tumor-Associated Macrophages and Macrophage-Related Immune Checkpoint Expression in Sarcomas. OncoImmunology.

[19] Bertucci F., Finetti P., Perrot D., Leroux A., Collin F., Le Cesne A., Coindre J.-M., Blay J.-Y., Birnbaum D., Mamessier E. (2017). PDL1 Expression Is a Poor-Prognosis Factor in Soft-Tissue Sarcomas. Oncoimmunology.

[20] Cassetta L., Kitamura T. (2018). Targeting Tumor-Associated Macrophages as a Potential Strategy to Enhance the Response to Immune Checkpoint Inhibitors. Front. Cell Dev. Biol..

[21] Kwai A., Nishikawa T., Kawasaki M., Tomatsuri S., Okamura N., Ogawa G., Hirakawa A., Shibata T., Nakamura T., Kakunaga S. Efficacy and Safety of Nivolumab Monothereapy in Patients with Unresectable Clear Cell Sarcoma and Alveolar Soft Part Sarcoma (OSARC TRIAL, NCCH1510): A Muticenter, Phase 2 Clinical Trial. Proceedings of the CTOS Annual Meeting.

[22] Mariuk-Jarema A., Koseła-Paterczyk H., Rogala P., Klimczak A., Wągrodzki M., Maksymiuk B., Rutkowski P. (2020). A Durable Complete Response to Immunotherapy in a Patient with Metastatic Alveolar Soft Part Sarcoma. Tumori.

[23] Hodge J.C., Pearce K.E., Wang X., Wiktor A.E., Oliveira A.M., Greipp P.T. (2014). Molecular Cytogenetic Analysis for TFE3 Rearrangement in Xp11.2 Renal Cell Carcinoma and Alveolar Soft Part Sarcoma: Validation and Clinical Experience with 75 Cases. Mod. Pathol..

[24] Aulmann S., Longerich T., Schirmacher P., Mechtersheimer G., Penzel R. (2007). Detection of the ASPSCR1–TFE3 Gene Fusion in Paraffin-Embedded Alveolar Soft Part Sarcomas. Histopathology.

[25] Brady O.A., Jeong E., Martina J.A., Pirooznia M., Tunc I., Puertollano R. (2018). The Transcription Factors TFE3 and TFEB Amplify P53 Dependent Transcriptional Programs in Response to DNA Damage. eLife.

[26] Morozov V.M., Massoll N.A., Vladimirova O.V., Maul G.G., Ishov A.M. (2008). Regulation of C-Met Expression by Transcription Repressor Daxx. Oncogene.

[27] Zhang J., Babic A. (2016). Regulation of the MET Oncogene: Molecular Mechanisms. Carcinogenesis.

[28] van Echten J., van den Berg E., van Baarlen J., van Noort G., Vermey A., Dam A., Molenaar W.M. (1995). An Important Role for Chromosome 17, Band Q25, in the Histogenesis of Alveolar Soft Part Sarcoma. Cancer Genet. Cytogenet.

[29] Sreekantaiah C., Li F.P., Weidner N., Sandberg A.A. (1991). Multiple and Complex Abnormalities in a Case of Alveolar Soft-Part Sarcoma. Cancer Genet. Cytogenet.

[30] Heidenblad M., Hallor K.H., Staaf J., Jönsson G., Borg Å., Höglund M., Mertens F., Mandahl N. (2006). Genomic Profiling of Bone and Soft Tissue Tumors with Supernumerary Ring Chromosomes Using Tiling Resolution Bacterial Artificial Chromosome Microarrays. Oncogene.

[31] Italiano A., Cardot N., Dupré F., Monticelli I., Keslair F., Piche M., Mainguené C., Coindre J.-M., Pedeutour F. (2007). Gains and Complex Rearrangements of the 12q13-15 Chromosomal Region in Ordinary Lipomas: The “Missing Link” between Lipomas and Liposarcomas?. Int. J. Cancer Res..

[32] Agaram N.P., Zhang L., Yun-Shao S., Singer S., Stevens T., Prieto-Granada C.N., Bishop J.A., Wood B.A., Swanson D., Dickson B.C. (2019). GLI1-Amplifications Expands the Spectrum of Soft Tissue Neoplasms Defined by GLI1 Gene Fusions. Mod. Pathol..

[33] Bagchi A., Mills A.A. (2008). The Quest for the 1p36 Tumor Suppressor. Cancer Res..

[34] Maris J.M., Weiss M.J., Guo C., Gerbing R.B., Stram D.O., White P.S., Hogarty M.D., Sulman E.P., Thompson P.M., Lukens J.N. (2000). Loss of Heterozygosity at 1p36 Independently Predicts for Disease Progression but Not Decreased Overall Survival Probability in Neuroblastoma Patients: A Children’s Cancer Group Study. J. Clin. Oncol..

[35] Zeng L., Fee B.E., Rivas M.V., Lin J., Adamson D.C. (2014). Adherens Junctional Associated Protein-1: A Novel 1p36 Tumor Suppressor Candidate in Gliomas (Review). Int. J. Oncol..

[36] Roubin R., Acquaviva C., Chevrier V., Sedjaï F., Zyss D., Birnbaum D., Rosnet O. (2012). Myomegalin Is Necessary for the Formation of Centrosomal and Golgi-Derived Microtubules. Biol. Open.

[37] Er T.-K., Su Y.-F., Wu C.-C., Chen C.-C., Wang J., Hsieh T.-H., Herreros-Villanueva M., Chen W.-T., Chen Y.-T., Liu T.-C. (2016). Targeted Next-Generation Sequencing for Molecular Diagnosis of Endometriosis-Associated Ovarian Cancer. J. Mol. Med..

[38] da Costa L.T., Dos Anjos L.G., Kagohara L.T., Torrezan G.T., De Paula C.A.A., Baracat E.C., Carraro D.M., Carvalho K.C. (2021). The Mutational Repertoire of Uterine Sarcomas and Carcinosarcomas in a Brazilian Cohort: A Preliminary Study. Clinics.

[39] Groisberg R., Roszik J., Conley A.P., Lazar A.J., Portal D.E., Hong D.S., Naing A., Herzog C.E., Somaiah N., Zarzour M.A. (2020). Genomics, Morphoproteomics, and Treatment Patterns of Patients with Alveolar Soft Part Sarcoma and Response to Multiple Experimental Therapies. Mol. Cancer Ther..

[40] Froimchuk E., Jang Y., Ge K. (2017). Histone H3 Lysine 4 Methyltransferase KMT2D. Gene.

[41] Maréchal A., Zou L. (2013). DNA Damage Sensing by the ATM and ATR Kinases. Cold Spring Harb Perspect Biol..

[42] Sun J., Wang Y., Xia Y., Xu Y., Ouyang T., Li J., Wang T., Fan Z., Fan T., Lin B. (2015). Mutations in RECQL Gene Are Associated with Predisposition to Breast Cancer. PLoS Genet..

[43] Lin J.J., Riely G.J., Shaw A.T. (2017). Targeting ALK: Precision Medicine Takes on Drug Resistance. Cancer Discov..

[44] Holla V.R., Elamin Y.Y., Bailey A.M., Johnson A.M., Litzenburger B.C., Khotskaya Y.B., Sanchez N.S., Zeng J., Shufean M.A., Shaw K.R. (2017). ALK: A Tyrosine Kinase Target for Cancer Therapy. Cold Spring Harb. Mol. Case Stud..

[45] Heist R.S., Shim H.S., Gingipally S., Mino-Kenudson M., Le L., Gainor J.F., Zheng Z., Aryee M., Xia J., Jia P. (2016). MET Exon 14 Skipping in Non-Small Cell Lung Cancer. Oncologist.

[46] Tang Z., Zhang J., Lu X., Wang W., Chen H., Robinson M.K., Cheng J., Tang G., Medeiros L.J. (2018). Coexistent Genetic Alterations Involving ALK, RET, ROS1 or MET in 15 Cases of Lung Adenocarcinoma. Mod. Pathol..

[47] Miller A.J., Levy C., Davis I.J., Razin E., Fisher D.E. (2005). Sumoylation of MITF and Its Related Family Members TFE3 and TFEB. J. Biol. Chem..

[48] Rota R., Ciarapica R., Miele L., Locatelli F. (2012). Notch Signaling in Pediatric Soft Tissue Sarcomas. BMC Med..

[49] Kolligs F.T., Bommer G., Göke B. (2002). Wnt/Beta-Catenin/Tcf Signaling: A Critical Pathway in Gastrointestinal Tumorigenesis. Digestion.

[50] Alshareef A., Gupta N., Zhang H.-F., Wu C., Haque M., Lai R. (2017). High Expression of β-Catenin Contributes to the Crizotinib Resistant Phenotype in the Stem-like Cell Population in Neuroblastoma. Sci. Rep..

[51] Lee C.-J., Wozniak A., Van Cann T., Timmermans I., Wellens J., Vanleeuw U., Briaire-de Bruijn I.H., Britschgi C., Bovée J.V.M.G., Zlobec I. (2021). Establishment of an Academic Tissue Microarray Platform as a Tool for Soft Tissue Sarcoma Research. Sarcoma.

[52] Cattoretti G., Bosisio F.M., Marcelis L., Bolognesi M.M. (2019). Multiple Iterative Labeling by Antibody Neodeposition (MILAN). Protoc. Exch..

[53] Bolognesi M.M., Manzoni M., Scalia C.R., Zannella S., Bosisio F.M., Faretta M., Cattoretti G. (2017). Multiplex Staining by Sequential Immunostaining and Antibody Removal on Routine Tissue Sections. J. Histochem. Cytochem..

[54] Loo P.V., Nordgard S.H., Lingjærde O.C., Russnes H.G., Rye I.H., Sun W., Weigman V.J., Marynen P., Zetterberg A., Naume B. (2010). Allele-Specific Copy Number Analysis of Tumors. Proc. Natl. Acad. Sci. USA.

[55] Scheinin I., Sie D., Bengtsson H., van de Wiel M.A., Olshen A.B., van Thuijl H.F., van Essen H.F., Eijk P.P., Rustenburg F., Meijer G.A. (2014). DNA Copy Number Analysis of Fresh and Formalin-Fixed Specimens by Shallow Whole-Genome Sequencing with Identification and Exclusion of Problematic Regions in the Genome Assembly. Genome Res..

[56] Mermel C.H., Schumacher S.E., Hill B., Meyerson M.L., Beroukhim R., Getz G. (2011). GISTIC2.0 Facilitates Sensitive and Confident Localization of the Targets of Focal Somatic Copy-Number Alteration in Human Cancers. Genome Biol..

[57] Sondka Z., Bamford S., Cole C.G., Ward S.A., Dunham I., Forbes S.A. (2018). The COSMIC Cancer Gene Census: Describing Genetic Dysfunction across All Human Cancers. Nat. Rev. Cancer.

[58] Cerami E., Gao J., Dogrusoz U., Gross B.E., Sumer S.O., Aksoy B.A., Jacobsen A., Byrne C.J., Heuer M.L., Larsson E. (2012). The CBio Cancer Genomics Portal: An Open Platform for Exploring Multidimensional Cancer Genomics Data. Cancer Discov..

[59] Kopanos C., Tsiolkas V., Kouris A., Chapple C.E., Albarca Aguilera M., Meyer R., Massouras A. (2019). VarSome: The Human Genomic Variant Search Engine. Bioinformatics.

[60] Freshour S.L., Kiwala S., Cotto K.C., Coffman A.C., McMichael J.F., Song J.J., Griffith M., Griffith O.L., Wagner A.H. (2021). Integration of the Drug–Gene Interaction Database (DGIdb 4.0) with Open Crowdsource Efforts. Nucleic Acids Res..

[61] Reimand J., Kull M., Peterson H., Hansen J., Vilo J. (2007). G:Profiler—a Web-Based Toolset for Functional Profiling of Gene Lists from Large-Scale Experiments. Nucleic Acids Res..

[62] Sidiropoulos K., Viteri G., Sevilla C., Jupe S., Webber M., Orlic-Milacic M., Jassal B., May B., Shamovsky V., Duenas C. (2017). Reactome Enhanced Pathway Visualization. Bioinformatics.

[63] Reimand J., Isser R., Voisin V., Kucera M., Tannus-Lopes C., Rostamianfar A., Wadi L., Meyer M., Wong J., Xu C. (2019). Pathway Enrichment Analysis and Visualization of Omics Data Using g:Profiler, GSEA, Cytoscape and EnrichmentMap. Nat. Protoc..

[64] Merico D., Isserlin R., Stueker O., Emili A., Bader G.D. (2010). Enrichment Map: A Network-Based Method for Gene-Set Enrichment Visualization and Interpretation. PLoS ONE.

