# Supplementary material for: Correlation of Immunological and Molecular Profiles with Response to Crizotinib in Alveolar Soft Part Sarcoma: An Exploratory Study Related to the EORTC 90101 “CREATE” Trial
Source: Int J Mol Sci. 2022 May 19;23(10):5689. doi: 10.3390/ijms23105689 (PMC9145625; doi:10.3390/ijms23105689)
Supplement: Supplementary file 1 [file ijms-23-05689-s001.zip › ijms-1732202-suppl/Supplementary Material 2.docx]

Supplementary Material 2: Representative images for MET expression in alveolar soft tissue sarcoma


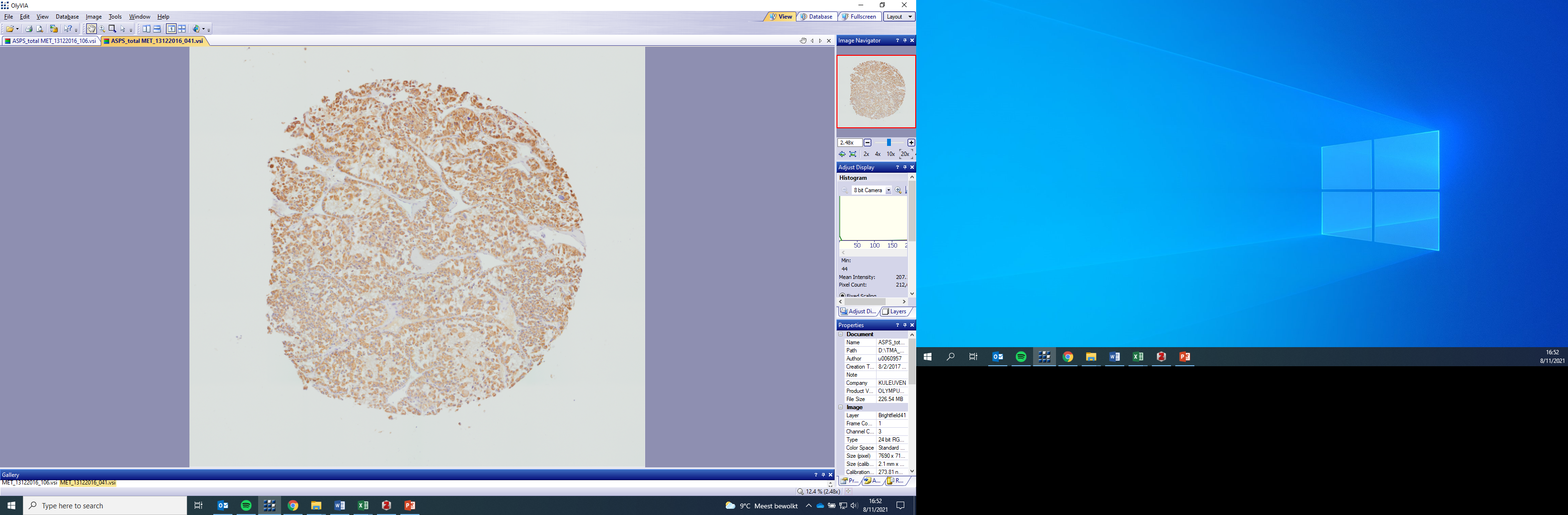


SeqID 054


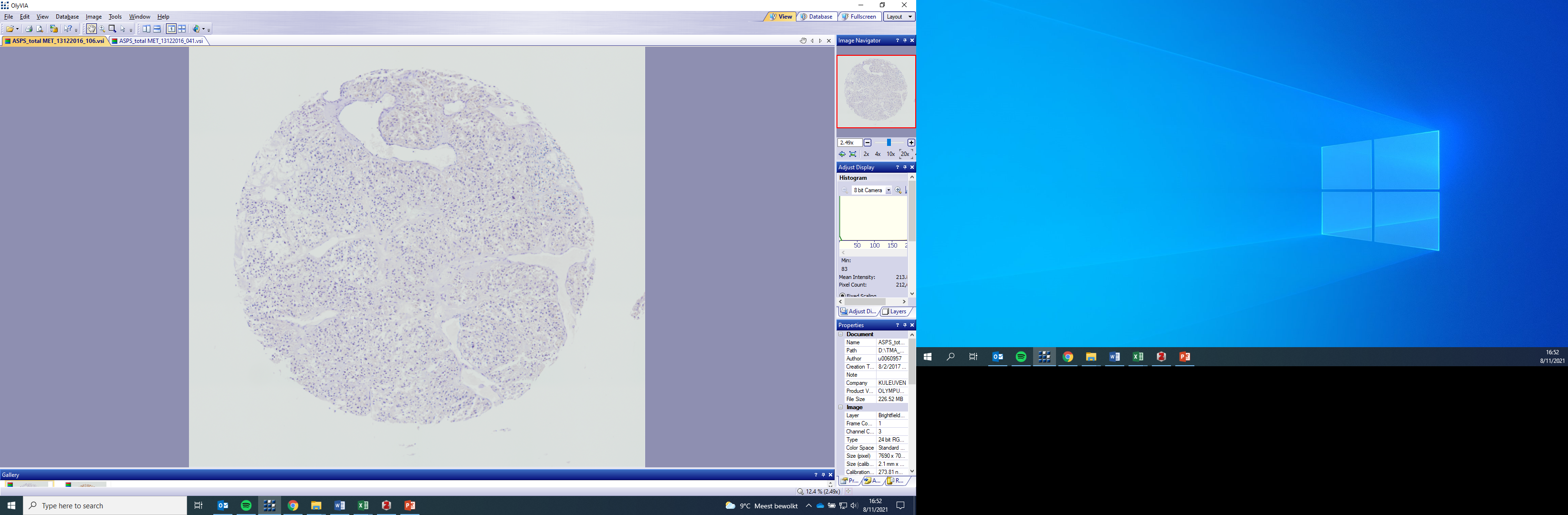


SeqID 109


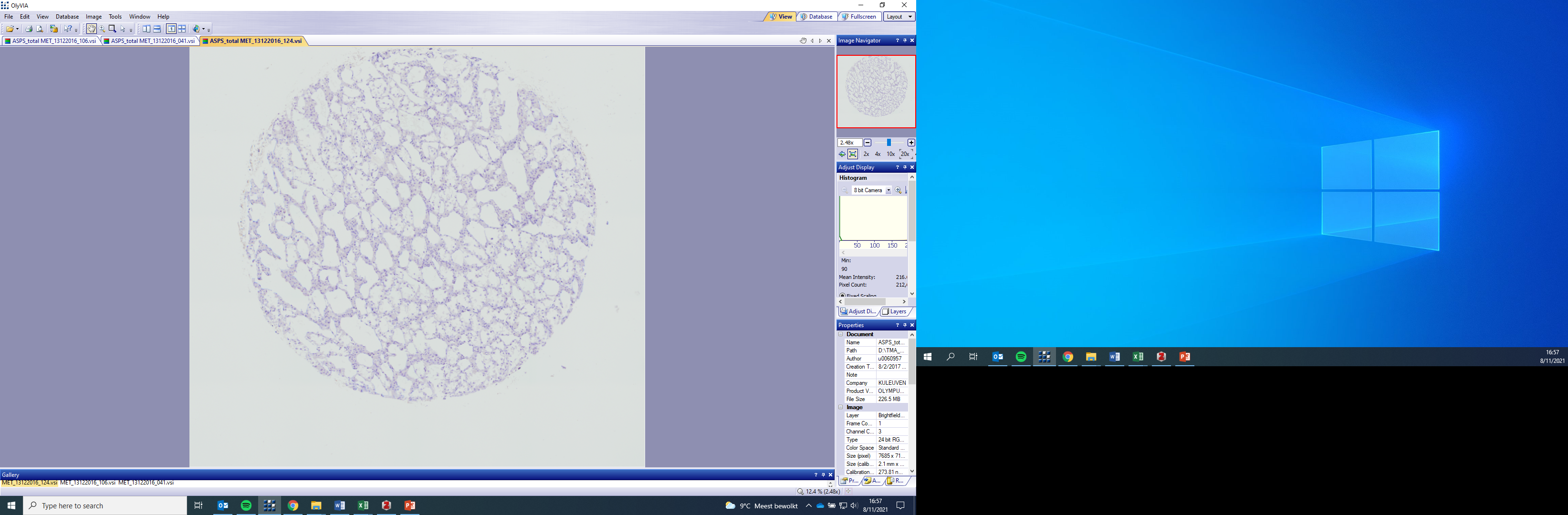


SeqID 133


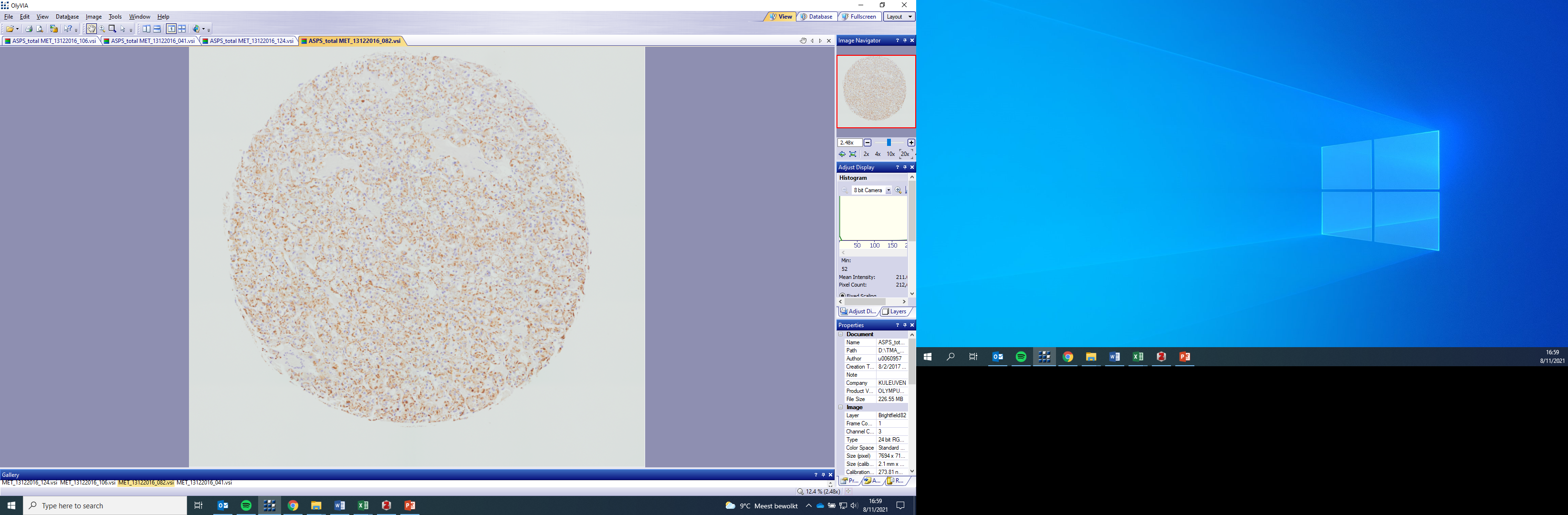


SeqID 089

MET overexpression

No MET expression
